# Decades of Night-Shift Work Induce Diurnal Disruption and Corneal Adaptations: Evidence from Pentacam Analysis

**DOI:** 10.3390/ijerph22040474

**Published:** 2025-03-23

**Authors:** Bence Lajos Kolozsvári, Éva Surányi, Zsuzsa Zakarné Aszalós, Vivien Lénárt, Reda Chaker, Géza Vitályos, Mariann Fodor

**Affiliations:** 1 Department of Ophthalmology, Gyula Petrányi Doctoral School of Allergy and Clinical Immunology, Faculty of Medicine, University of Debrecen, Nagyerdei krt. 98, 4032 Debrecen, Hungary; kolozsvari.bence@med.unideb.hu (B.L.K.); suranyi.eva@med.unideb.hu (É.S.); azsuzsa20@gmail.com (Z.Z.A.); lenart.vivien@med.unideb.hu (V.L.); redachaker97@gmail.com (R.C.); 2 Department of Pediatric Dentistry and Orthodontics, Faculty of Dentistry, University of Debrecen, 4012 Debrecen, Hungary; vitalygdr@gmail.com

**Keywords:** cornea, diurnal changes, sleep deprivation, night shift, anterior segment of the eye, Scheimpflug parameters, pachymetry, keratometry

## Abstract

We aimed to determine the effects of night-shift work on corneal parameters in thirty-five healthy individuals (24–59 years) in a retrospective cohort study. Among them, 12 hospital nurses regularly worked two shifts, spending a third of their nights awake, whereas 23 age-matched controls never worked shifts and slept regularly. Measurements were performed at least five times within 12 h. We analyzed the keratometric parameters of the corneal front (F) and back (B) surfaces, including the refractive power in the flattest and steepest axes (K1, K2), astigmatism (Astig); and corneal pachymetry (Pachy) at the thinnest corneal point and pupil center, volume relative to the 10 mm corneal diagonal (Vol D10); and surface variance index (ISV). A multilevel mixed-effects linear regression adjusted for age was applied to 905 measurements. All parameters exhibited significant periodic fluctuations (*p* ≤ 0.005). The two groups also showed significantly different periodic fluctuations (*p* ≤ 0.008), except in K1B and AstigB. K1/K2 (F and B), AstigF, Pachy, and ISV differed significantly (*p* < 0.0001). Surprisingly, prolonged night shift work did not increase the ISV, and no evidence of age-related corneal thinning was observed. Long-term night-shift exposures change various corneal parameters, reflecting both concomitant and adaptive effects. This study highlights the impact of consistent sleep deprivation on corneal properties, warranting further research into understanding the long-term effects of night-shift work.

## 1. Introduction

Human physiology adheres to a 24 h circadian cycle maintained by conserved and self-regulating processes. The diurnal cycle is governed by day–night alternations. Vision, intricately and closely associated with fluctuations in light intensity throughout the day, functions rhythmically [1]. Modern lifestyles, including extensive artificial light during the night and sleep deprivation, challenge these rhythms and remain to be elucidated [1].

The circadian rhythm helps to maintain the physiological functions in humans [2,3,4]. Disruptions in the circadian rhythm, such as those caused by sleep disorders, night shift work, and jet lag, can negatively affect physiological functions, including ocular health. It has been observed that people who work night shifts are at a higher risk of developing ocular disorders, such as dry eye syndrome, glaucoma, and cataracts [5,6,7,8,9]. This may be due to a complex mechanism, including the decreased production of melatonin, which has antioxidant and anti-inflammatory effects [10,11,12,13].

The cornea is a transparent avascular structure that refracts and focuses light onto the lens, thereby contributing fundamentally to visual perception. Additionally, it acts as a physical barrier equipped with a protective tear film and sensory nerves that help detect pressure and pain changes, facilitating the eye’s protection from external trauma, dust, and microorganisms [7,14]. The cornea has a unique anatomical structure that provides it with strength, elasticity, and transparency. These properties are important to fulfill its refractive function. Measuring basic corneal parameters, such as the thickness, curvature, and topography, provides crucial information about corneal health and function.

Around 21% of employees work in shifts [15,16]. Night shift work is inevitable in nursing [5,17]. Within the scientific literature, the significant impact of night-shift-induced sleep deprivation on corneal characteristics remains a largely neglected research area [18]. We focused on the population of shift-working nurses—with validated work-schedule data—because they face unique challenges, including disrupted sleep patterns and circadian rhythm disturbances. Since this population requires sustained high-quality vision during all-night shifts, assessing their corneal health is essential for informing targeted health interventions. To the best of our knowledge, no study has addressed the diurnal variation in corneal parameters for long-time night-shift nurses covering the entire day. This study aims to bridge the gap by employing the Pentacam—renowned for its outstanding repeatability [19]—to evaluate diurnal corneal variations among healthy subjects working night shifts as nurses versus those not engaged in night-shift duties. We aim to investigate whether chronic, long-lasting night-shift work accelerates the age-related thinning of the cornea and causes significant changes in its optical properties. Specifically, we hypothesize that if long-term night shift work has a detrimental effect on corneal health, it will lead to measurable alterations, such as accelerated thinning and other changes in optical parameters. Should our results indicate that chronic night-shift work poses risks to corneal integrity and visual quality, these findings could inform interventions to protect the ocular health of night-shift workers.

## 2. Subjects and Methods

Our retrospective observational study included 35 healthy subjects of European or Arabian origin, each with 0.00 logMAR distance visual acuity, a low refractive error (lower than 3.0 diopters [D]), a normal tear meniscus, and no ophthalmological disorders. All participants, who were exclusively medical staff or employees, provided written informed consent in accordance with the Declaration of Helsinki. The University of Debrecen’s Research Ethics Committee then approved the study protocol (DE RKEB/IKEB: 5418-2020).

Exclusion criteria comprised any refractive error exceeding 3.0 D (encompassing both myopia or hyperopia); the presence of active inflammatory or infectious diseases, systemically or ocularly; dry eye conditions; current ongoing treatment with systemic or local medications; the use of eye drops; contact lens usage; a history of ocular surgery; any lens or retinal irregularities observed during biomicroscopic examination; a history of chemical injury or delayed epithelial recovery; pregnancy or lactation; and any sleep disorders. Prior to the ophthalmologic assessment, three high-resolution Pentacam (Pentacam AXL, Oculus Optikgeräte GmbH, Wetzlar, Germany, software version 1.25r15) images were captured for each participant’s eyes. Should any image exhibit distortion (e.g., blinking), missing data, or quality other than “OK”, the image was retaken.

Current and lifetime night-shift work information was obtained. Participants were divided into two groups: “night shift working nurses” and “non-nurses”, the latter group included day workers who served as controls. We calculated the duration (number of years working night shifts), and frequency (average number of night shifts per month) of night-shift work. Typically, the nurses worked two shifts, spending approximately one-third of their nights awake and on duty. In contrast, control subjects never engaged in shift work and maintained regular nighttime sleep, except when nocturnal measurements were required for this study. Each participant underwent assessments every 3–4 h over a 24 h period, with a minimum of 5 measurements per day, as described in one of our earlier studies [20]. The study offered two measurement protocols for the participants: (1) six measurements over 24 h without sleep (scheduled at 05:30–08:30, 09:00–11:30, 12:30–15:00, 16:00–18:30, 20:00–23:30, and 00:30–04:30); or (2) five measurements during each of two 12 h intervals, scheduled no more than one week apart. Most nurses opted for the latter protocol. Specifically, during the “morning shift,” measurements occurred at approximately 06:30, 09:00, 12:30, 15:00, and 17:30. During the “night shift” (i.e., for sleep-deprived subjects), measurements were conducted around 18:00, 21:00, 00:00, 03:00, and 05:30. Additionally, motivated “non-nurse” participants completed both protocols to ensure full 24 h coverage. Participants continued with normal activities between measurements while refraining from alcohol and other drugs. The sleep-deprived group remained in well-lit conditions with their eyes open and were not permitted to sleep between assessments; they either worked at the study site or, in the case of controls, participated in work meetings.

From each session, all three Pentacam images’ data were averaged to yield session-level values. Subsequently, the following parameters were exported from the Pentacam into Microsoft Excel (2502 build version 16.0.18526.20168 64 bites, Microsoft Corp, Redmond, Washington): Holladay equivalent keratometry readings for the flat (K1) and steep (K2) meridians of both the anterior (F) and posterior (B) surfaces; corneal astigmatism for the anterior (Astig F) and posterior (Astig B) surfaces; corneal thickness at the thinnest point (Pachy Min) and at the pupil center (Pachy Pupil); corneal volume within a 10 mm diameter centered on the anterior corneal apex (Vol D 10 mm); and the index of surface variation (ISV).

### Statistical Methods

One eye from each participant was randomly chosen for evaluation. Each set of measurement sessions per participant received a distinct and unique session identifier. For descriptive intents, the observed limits (minimum and maximum) of the outcome variables were utilized to calculate the observed ranges of within-subject diurnal change. The minimum, maximum, and range were summarized across participants using standard statistical methods by subject group (nurses involved in night shift work versus non-nurses).

To evaluate diurnal variation using regression modeling, we derived trigonometric predictors based on the sine and cosine of appropriate multiples of 2π, 4π, and 6π, which were generated from the exam time and expressed as the fractional time of day. These predictors were incorporated as fixed-effect terms in the interaction with the subject group identifier and adjusted for age within a multilevel mixed-effects linear regression model featuring a random intercept varying independently across subjects and sessions. Diurnal variation was then assessed by subject group in terms of the minimum and maximum outcomes predicted by the fixed-effects component for a subject at the sample mean age in that given group, indicating the times when these extremes occurred and the estimated difference between the modeled maximum and minimum, reported as a point estimate with its *p*-value and 95% confidence interval. The estimated values of age-adjusted between-group differences were specifically calculated for midnight, 6 a.m., noon, and 6 p.m. and described using similar parameters. Trends were illustrated via scatter plots of the fitted outcome values against the time of day, with subject- and session-level variability removed (referred to as shifted fitted values), and the underlying backbone of the trend at subject-group-specific sample mean ages was superimposed. A *p*-value <0.05 was considered indicative of a significant difference.

Trigonometric regression was used to account for periodicity in the data. Multiples of 4π and 6π (in addition to 2π) were applied to avoid forcing a single-peak, single-trough fit (such a fit could still occur naturally if the data support it). Multilevel mixed-effects modeling was used since the observations were not independent. An interaction with the subject group identifier was included to allow for different patterns across subject groups. The four chosen time points were arbitrary; the goal was to cover the entire day while balancing between spacing that was too sparse or too dense.

## 3. Results

The study included 35 participants, with 23 non-nurses (control group) and 12 night-shift-working nurses. The mean age of the control group was 35.3 years (SD = 10.03), while the mean age of the nurses was 43.1 years (SD = 12.6). There was no significant difference in age between the two groups (*p* = 0.054). The age range of the participants was from 24 to 59 years. From each participant, one randomly selected eye was analyzed. Overall, there were 19 women (54%) and 16 men; 17 were right eyes and 18 were left eyes. Participants were predominantly female (54%). The male-to-female ratio among nurses was 1:11, whereas among non-nurses, it was 8:15. Results with and without an adjustment for sex were systematically compared, but no significant effect of such adjustment was observed. The nurses had been on a night-shift schedule for an average of 19 years (SD: 13.6; range: 4–40 years) prior to enrollment in the study. A total of 905 measurements revealed a significant periodic fluctuation in all analyzed parameters (*p* ≤ 0.005).

### 3.1. Differences Between the Groups (Nurses vs. Non-Nurses) Regarding the Keratometry Values Including Astigmatism

Table 1 shows the unadjusted mean measurement results, revealing that K1 and K2 on the anterior surface were significantly lower in the control group than in the nurse group (*p* < 0.0001). On the posterior surface, K1 and K2 were significantly lower in the night shift group than in the non-night shift group (*p* < 0.0001). The front surface astigmatism was significantly higher in the nurses group than in the controls (*p* < 0.0001), but there was no significant difference in the back surface astigmatism between the groups (*p* = 0.515).

There were significant differences between the nurses and non-nurses in K2F and Astig F at the shift change at 06:00 and 18:00 (*p* = 0.046 and *p* = 0.017, respectively) and also at midnight in K2F (*p* = 0.039) (Table 1).

**Figure 1:**
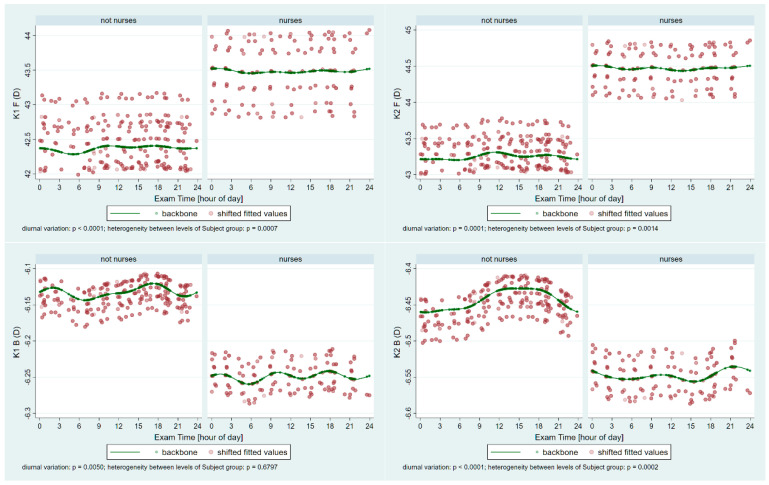
Periodic fluctuations of the flat (**K1**)—and steep-keratometry (**K2**) on the front (**F**) and the back (**B**) surface of the cornea in the control group (not nurses) and the nurses. Significant periodic fluctuations can be observed in all parameters (*p* ≤ 0.005), and significant heterogeneity is present between the groups in all parameters (*p* ≤ 0.001), except K1B (*p* = 0.6797). The higher front corneal surface keratometry values (more convex) in the nurses’ group are compensated for with the lower (more concave) values on the back surface.

**Figure 2:**
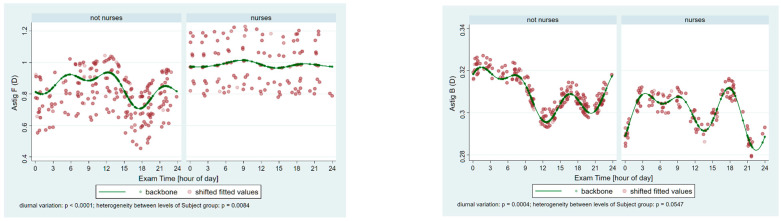
Periodic fluctuations in astigmatism (Astig) on the front (**F**) and the back (**B**) surface of the cornea in the control group (not nurses) and the nurses. Significant periodic fluctuations can be observed on both surfaces (*p* ≤ 0.0004). Significant heterogeneity is present between the groups in the case of the front surface (*p* = 0.0084), but not in the back surface (*p* = 0.0547).

Figure 1 shows periodic fluctuations based on age-adjusted modeling of the flat (K1)- and steep-axis keratometry (K2) on the front (F) and the back (B) surface of the cornea in the two groups. Significant periodic fluctuations can be observed in all parameters (all *p* ≤ 0.0001; except K1B *p* = 0.005), and significant heterogeneity is present between the groups in all parameters (*p* ≤ 0.001), except K1B (*p* = 0.6797). The higher front corneal surface keratometry values (more convex) in the nurses’ group are in line with the lower (more concave) values on the back surface. Figure 2 presents the significant periodic fluctuations of AstigF and AstigB (*p* ≤ 0.0004) and significant heterogeneity between the groups in AstigF (*p* = 0.0084), but not in the back surface (*p* = 0.0547).

### 3.2. Differences Between the Groups Regarding Corneal Pachymetry Values and Index of Surface Variance (ISV)

**Figure 3:**
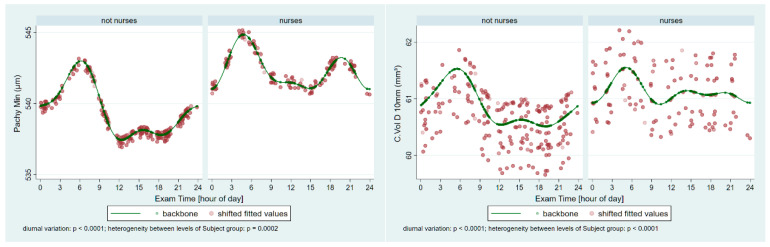
Periodic fluctuations in the corneal thickness at the thinnest point of the cornea (Pachy Min) and the volume of the cornea in a diameter of 10 mm centered on the anterior corneal apex (Vol D 10 mm) in the control group (not nurses) and the nurses (age-adjusted data). Significant periodic fluctuations can be observed (both *p* ≤ 0.0001), and significant heterogeneity is present between the groups (*p* = 0.0002 and *p* < 0.0001, respectively).

**Figure 4:**
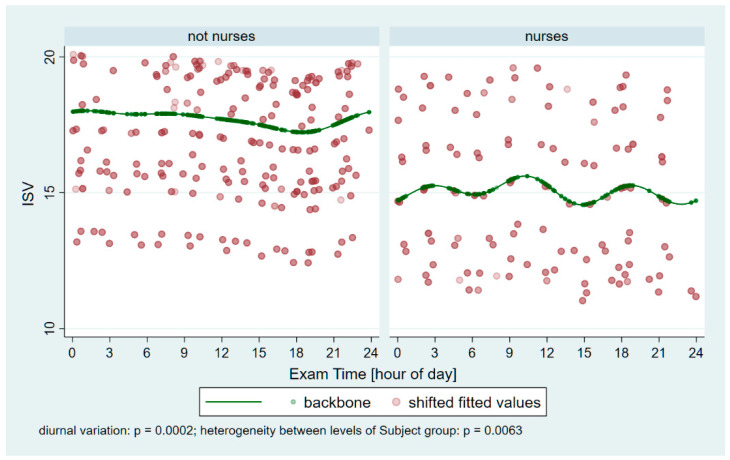
Periodic fluctuations in the index of surface variance (ISV) in the control group (not nurses) and the nurses. Significant variation can be observed (*p* = 0.0002), and significant heterogeneity is present between the groups (*p* = 0.0063).

The corneal thicknesses at the thinnest point of the cornea (Pachy Min) and at the pupil’s center (Pachy Pupil), as well as the volume of the cornea in a diameter of 10 mm centered on the anterior corneal apex (Vol D 10 mm), were analyzed and compared between the two groups. Table 2 shows the unadjusted means, as well as the model-estimated minimum and maximum values and the time points of these extremes, for the pachymetric and ISV data. Our analysis revealed that Pachy Min and also Pachy Pupil were significantly lower in the night-shift group than in the non-night-shift group (*p* = 0.041 and *p* = 0.007, respectively), but the corneal volume in the central 10 mm failed to reach a significant difference (*p* = 0.317). For all pachymetric parameters with age-adjustment and also for ISV, the significance of periodic fluctuations is *p* ≤ 0.0002 (Figure 3 and Figure 4). The heterogeneity between the two groups is *p* ≤ 0.0063 for all parameters. 

The Index of Surface Variance data (Table 2 and Figure 4), which reflects the irregularity of curvature of the anterior corneal surface, showed a significant unadjusted difference between night-shift workers and non-night-shift workers (*p* = 0.039). However, the ISV value was no longer found to be significantly lower in night-shift workers after an adjustment for age.

ISV was the only outcome variable found to be significantly affected by age: older subjects had lower values (estimated change: −2.17 [95%CI: −4.14 to −0.20, *p* = 0.031] associated with +10 years of age, adjusted for subject group and trigonometric predictors).

## 4. Discussion

Our study aimed to investigate how long-term night-shift work, particularly its diurnal disruption, impacts the cornea and overall ocular health. In modern society, as many as 21% of all employees work in shifts [15]. Growing evidence indicates that night-shift work adversely affects health and organ functions, and these effects may be more significant with long-term night-shift exposure [16]. To the best of our knowledge, this is the first comprehensive study to assess corneal parameters in long-term night-shift nurses over a 24 h period. Our research revealed significant differences between shift workers and non-shift workers concerning the periodic fluctuations of many corneal parameters, including the keratometry, pachymetry, volumes, and surface variance.

Working night shifts is associated with disruptions to the diurnal rhythm, a lack of sleep, and sleep deprivation. Sleep deprivation is a common public health problem and can cause various diseases, such as diabetes, obesity, atrial fibrillation, and vascular diseases, including coronary heart disease [16,21]. Water intake during the night shift is typically lower [22], which can lead to dehydration and potentially serious consequences. Sleep deprivation increases the risk of ocular diseases, including dry eye and corneal epithelial lesions [23,24].

Our measurements revealed significant periodic fluctuations in all tested corneal parameters (K1 F/B, K2 F/B, Astig F/B, Pachy Min/Pupil, Vol D10, and ISV). This finding is consistent with an earlier observation examining the effect of one night (short-term) of sleep deprivation on overnight corneal changes, although that study did not focus on night-shift nursing work [20]. We observed significant heterogeneity between shift workers and the control group in all parameters, except K1B and Astig B. This result supports the idea that the inner surface of the cornea—less exposed to environmental changes and disruptions caused by night-shift schedules—is more stable and less prone to fluctuations than the front surface.

The cornea provides most of the eye’s refractive power; this focusing capacity depends largely on the cornea’s fundamental characteristics. Even a small change in the corneal thickness can be associated with altered keratometry, causing a change in function. Age-dependent corneal thinning was observed in earlier studies [25]. Previous research indicates that, in mice, sleep deprivation damages the barrier and pump functions of the corneal endothelium, leading to increased oxidative stress and mitochondrial dysfunction. As a result, these changes can lead to corneal endothelial cell dysfunction, corneal edema, and increased corneal thickness [23]. Notably, an increased cornea thickness is a hallmark of endothelial cell functional impairment [23].

Our study revealed that corneal thickness (PachyMin, Pupil, and Vol D 10) was not lower in the night-shift group compared to the non-night-shift group at the same age; however, the average age of the nurses was higher and, consistently with earlier studies, the age-unadjusted corneal thickness parameters PachyMin and Pupil were lower. The long-term night shift might cause endothelial cell dysfunction and consequently corneal edema, and the age-dependent thinning might also be modified. In addition to endothelial functions, the body’s hydration status also affects the thickness of the cornea. During night shifts, water intake typically decreases [22], potentially leading to dehydration and reducing the overall body water content. Such changes, even if minor, can affect corneal biomechanical properties [26]. In the future, investigating both the number and quality of corneal endothelial cells in night-shift nurses, as well as the regenerative capacity and adaptability mechanisms of the cornea, could yield valuable insights into the differences between shift workers and non-shift workers.

The only study analyzing the corneal biomechanical behavior among night-shift workers found that night shifts do not independently affect Corvis corneal biomechanical indices and properties [18]. Furthermore, significant correlations were presented between the Corvis Deformation Amplitude ratio and night-shift period, as well as between the Integrated Radius index and the number of monthly night shifts. However, the leading corneal biomechanics indices did not show any significant differences or correlations [18]. Moreover, in that study, the thinnest point of the cornea (PachyMin), as measured using a Pentacam, showed no significant difference between nurses and non-nurses of the same age, consistent with our findings [18]. The participants in Shirzadi et al. were much younger than ours (mean age 28.4 vs. 43.1 years) and had worked night shifts for a significantly shorter period (3 years vs. 19 years). Furthermore, only one measurement was taken during the entire day. These factors may explain why that study did not detect any clinically relevant biomechanical differences between night-shift workers and controls [18]. Our findings suggest that the observed pachymetric deviations and the heterogeneity between the two groups are consequences of regular, long-term night-shift work and may affect corneal biomechanical properties, which warrants further investigation.

The keratometry values of the cornea are crucial for proper vision and exhibit a pronounced diurnal rhythm [20]. Posterior topographic indices remain more stable during the day [20], indicating that the front corneal surface is more susceptible to environmental impacts, while the back surface experiences fewer external influences. When the cornea swells due to edema, its refractive power decreases. This is because the water is able to displace some of the tissue volume [27]. Variations in the corneal thickness over time and amplitude among night-shift workers may explain their higher keratometric measurements. The degree of astigmatism is also higher on the anterior surface in the night-shift group. The timing of maximum astigmatism also differed between the groups, indicating distinct periodic fluctuations. No significant difference in posterior-surface astigmatism was observed between the night-shift and control groups, suggesting that the cornea’s posterior surface is more stable and less easily influenced by external factors. This observation is especially significant, as the posterior surface accounts for approximately 31% of the astigmatism observed in the anterior cornea [28].

The Index of Surface Variance reflects the irregularity of curvature of the anterior corneal surface. Notably, the ISV was significantly lower in night-shift workers (*p* = 0.0394), indicating that, on average, their corneal surface remained more regular throughout the day than that of non-night-shift workers. This observation is in line with our experience that several control participants reported symptoms of dry eyes between 1:00 a.m. and 5:00 a.m., and their Pentacam measurements often had to be repeated several times to obtain valid data. These findings highlight the adaptational potential of night-shift workers’ corneas. Such adaptation might involve modifications in corneal epithelial mitosis and clock gene expression, alterations in epithelial lipid accumulation and microvilli morphology, and changes in tear production, including variations in tear osmolarity.

A major strength of our study is the large number of measurements collected across the entire 24 h period, ensuring accurate data yields, and encompassing a wide age range (24 to 59 years). Participants remained on site late at night or until morning for night measurements to obtain “true” values, thus avoiding extrapolated or missing data. Another novel aspect is that, for the first time, a comprehensive evaluation was carried out on night-shift workers for a unique chance to examine the relationship between night-shift work and human ocular health. This approach provides valuable information for further studies and potential preventive modalities. The main finding of our investigation is that, although long-term night-shift exposure did not lead to a significant decrease in central pachymetry, we found evidence that it may induce an increase in selected keratometry parameters. Due to the adaptation, throughout the 24 h period, one of the quality indices of the ocular surface (ISV) remained better among night-shift workers than in controls, who never worked shifts nor were used to being up all night long. Our findings provide novel insights into ocular surface homeostasis and expand our knowledge of corneal alterations caused by long-term night-shift work.

Limitations of this study include the exclusion of several systemic and physiological factors, such as the participants’ weight, hydration status, menstrual cycle, diet, smoking habits, coffee consumption, alcohol intake from the previous day, duration of exposure to indoor electric lights, ambient air humidity, and eyelid closure duration or sleep patterns. Nonetheless, given that participants were actively working and were commuting during the study, significant alcohol consumption was unlikely. All measurements were conducted in the same room using identical equipment in complete darkness. A more comprehensive investigation of these factors might have provided further valuable insights. Moreover, none of the sleep-deprived participants were maintained in dark or low-blue-light conditions, nor were they allowed to close their eyes, which might have elucidated the influence of varying melatonin levels on corneal circadian fluctuations. Unidentified and unmeasured confounding factors may still exist and affect the cornea. This cohort included mostly people of European descent, which limits the generalizability to other ethnicities. The endothelial cell count was not measured, although it could have provided valuable information. Although there was no statistically significant difference in age between the two groups, the nurses tended to be older. Intriguingly, this suggests that the older night-shift workers’ corneas did not thin with age as one might expect, an observation requiring further research. In spite of these limitations, our findings underscore that anterior and posterior keratometry, pachymetry, and the ISV index exhibit significant differences between nurses and non-nurses. The light–dark cycle plays a crucial role in renewing the corneal epithelium. Furthermore, exposure to constant light, persistent darkness, or sleep deprivation can alter the diurnal pattern of corneal epithelial miosis and clock gene expression [1]. Globally, sleep deficiency and deprivation have emerged as significant public health concerns, impacting various aspects of health, including ocular surface disorders [9]. Our comprehensive evaluation of long-term night-shift workers, who often work for decades awake at night and asleep during the day, reveals a modified picture of ocular surface homeostasis. Further investigations are required to elucidate the practical implications of our findings, particularly with regard to potential clinical applications and work scheduling strategies. The ocular effects of the night-shift work have not been thoroughly studied; thus, our findings warrant further exploration. Future studies could systematically assess the duration and frequency of night-shift work using objectively documented information. This is because in our study, only the past five years of documented data on the shift work were precise; details from earlier years were largely speculative.

Our findings highlight the potential impact of night-shift work on the corneal surface and suggest that further investigations are needed to understand the underlying mechanisms. Determining the tolerable frequency and duration of night-shift work warrants further study. Our findings have public health implications suggesting a lack of evidence in this field. These results may also be relevant for various professions, underscoring the importance of monitoring the ocular health of those who work night shifts. Further studies are required to clarify the underlying mechanisms behind these observations.

## 5. Conclusions

Our results showed that long-term night-shift work (average of 19 years) has a significant influence on the human cornea. However, significant negative consequences could not be detected, highlighting the importance of adaptation mechanisms. Despite the high prevalence of shift work, relatively few studies focus on this population. Further research is essential to confirm our findings, explore adaptive mechanisms, investigate potential impacts on vision, and reveal possible preventive modalities.

## Figures and Tables

**Table 1:** Periodic fluctuations in keratometry parameters in nurses (N = 12) and non-nurse subjects (N = 23) measured with a Pentacam AXL (K1 = flat-axis keratometric value in diopters (D) on anterior (F) and posterior (B) corneal surface; K2 = steep-axis keratometric value in diopters (D) on anterior (F) and posterior (B) corneal surface; Astig = corneal astigmatism of the front (F) and the back (B) surface; SD = standard deviation; *p* = *p*-value). Mean measurement results: unadjusted means of all observations of all group members; estimated minimum and maximum values and time: minimum and maximum of model-fitted values and time of day when observed; estimated significant differences specific to the time of day: modeling estimates of nurses vs non-nurses, difference at a pooled mean age and 00:00, 06:00, 12:00, or 18:00 h.

	NURSES			NON-NURSES			Estimated Significant Differences Specific to Time of Day
	Mean Measurement Result (SD)	Estimated Minimum Value and Time	Estimated Maximum Value and Time	Mean Measurement Result (SD)	Estimated Minimum Value and Time	Estimated Maximum Value and Time	
**K1 F**	43.42 (1.38)	43.46, 05:50	43.52, 00:52	42.41 (1.62)	42.28, 05:07	42.4, 10:28	
**K2 F**	44.41 (1.34)	44.43, 13:40	44.51, 00:41	43.20 (1.60)	43.2, 05:32	43.31, 11:37	at 00:00 h *p* = 0.039 at 06:00 h *p* = 0.046
**K1 B**	−6.238 (0.239)	−6.259, 05:07	−6.242, 18:02	−6.131 (0.257)	−6.14, 06:44	−6.122, 18:02	
**K2 B**	−6.537 (0.254)	−6.555, 16:13	−6.535, 21:37	−6.444 (0.298)	−6.46, 00:41	−6.427, 14:23	
**Astig F**	0.990 (0.330)	0.966, 15:01	1.014, 09:14	0.751 (0.505)	0.707, 17:42	0.937, 12:08	at 18:00 h *p* = 0.017
**Astig B**	0.301 (0.084)	0.2916, 13:20	0.3114, 18:07	0.312 (0.129)	0.296, 12:18	0.322, 01:11	

**Table 2:** Periodic fluctuations in pachymetric and volumetric corneal parameters and index of surface variation in nurses (N = 12) and non-nurse subjects (N = 23) measured with a Pentacam AXL. (Pachy Min: corneal thickness at the thinnest point of the cornea (µm); Pachy Pupil: corneal thickness at the pupil’s center (µm); Vol D10 mm: volume of the cornea in a diameter of 10 mm, centered on the anterior corneal apex (mm^3^), ISV = index of surface variation; SD = standard deviation; *p* = *p*-value). Mean measurement results: unadjusted means of all observations of all group members; estimated minimum and maximum values and time: minimum and maximum of model-fitted values and time of day when observed.

	NURSES			NON-NURSES		
	Mean Measurement Result (SD)	Estimated Minimum Value and Time	Estimated Maximum Value and Time	Mean Measurement Result (SD)	Estimated Minimum Value and Time	Estimated Maximum Value and Time
**Pachy Min**	543.1 (30.5)	541.1, 15:01	543.5, 02:46	545.7 (37.9)	537.5, 12:53	542.5, 07:24
**Pachy Pupil**	545.2 (31.1)	543.3, 15:27	547.1, 04:23	548.8 (38.0)	540.4, 12:32	545.5, 07:08
**Vol D10**	61.15 (3.25)	60.9, 10:13	61.55, 05:07	61.06 (3.52)	60.52, 19:31	61.52, 05:07
**ISV**	15.01 (3.71)	14.61, 22:12	15.6, 09:57	16.82 (7.72)	17.23, 18:05	18.00, 00:21

## Data Availability

The datasets analyzed during the current study are available from the corresponding author upon reasonable request.
